# The Impact of Variational Primary Collaterals on Cerebral Autoregulation

**DOI:** 10.3389/fphys.2018.00759

**Published:** 2018-06-19

**Authors:** Zhen-Ni Guo, Xin Sun, Jia Liu, Huijie Sun, Yingkai Zhao, Hongyin Ma, Baofeng Xu, Zhongxiu Wang, Chao Li, Xiuli Yan, Hongwei Zhou, Peng Zhang, Hang Jin, Yi Yang

**Affiliations:** ^1^Department of Neurology, The First Hospital of Jilin University, Changchun, China; ^2^Department of Neurology, Clinical Trial and Research Center for Stroke, The First Hospital of Jilin University, Changchun, China; ^3^Shenzhen Institutes of Advanced Technology, Chinese Academy of Sciences, University Town of Shenzhen, Shenzhen, China; ^4^Cadre Ward, The First Hospital of Jilin University, Changchun, China; ^5^Department of Neurosurgery, The First Hospital of Jilin University, Changchun, China; ^6^Department of Radiology, The First Hospital of Jilin University, Changchun, China

**Keywords:** dynamic cerebral autoregulation, cerebrovascular stenosis, anterior communicating artery, posterior communicating artery, digital subtraction angiography

## Abstract

The influence of the anterior and posterior communicating artery (ACoA and PCoA) on dynamic cerebral autoregulation (dCA) is largely unknown. In this study, we aimed to test whether substantial differences in collateral anatomy were associated with differences in dCA in two common types of stenosis according to digital subtraction angiography (DSA): either isolated basal artery and/or bilateral vertebral arteries severe stenosis/occlusion (group 1; group 1A: with bilateral PCoAs; and group 1B: without bilateral PCoAs), or isolated unilateral internal carotid artery severe stenosis/occlusion (group 2; group 2A: without ACoA and with PCoA; group 2B: with ACoA and without PCoAs; and group 2C: without both ACoA and PCoA). The dCA was calculated by transfer function analysis (a mathematical model), and was evaluated in middle cerebral artery (MCA) and/or posterior cerebral artery (PCA). Of a total of 231 non-acute phase ischemic stroke patients who received both dCA assessment and DSA in our lab between 2014 and 2017, 51 patients met inclusion criteria based on the presence or absence of ACoA or PCoA, including 21 patients in the group 1, and 30 patients in the group 2. There were no significant differences in gender, age, and mean blood pressure between group 1A and group 1B, and among group 2A, group 2B, and group 2C. In group 1, the PCA phase difference values (autoregulatory parameter) were significantly higher in the subgroup with patent PCoAs, compared to those without. In group 2, the MCA phase difference values were higher in the subgroup with patent ACoA, compared to those without. This pilot study found that the cross-flow of the ACoA/PCoA to the affected area compensates for compromised dCA in the affected area, which suggests an important role of the ACoA/PCoA in stabilizing cerebral blood flow.

## Introduction

Primary collaterals include the anterior and posterior communicating arteries (ACoA and PCoA), are crucial to maintaining adequate cerebral perfusion, and their anatomic variants could pose a challenge for individuals during ischemic demand. Several studies have reported the influence of the ACoA and PCoA on cerebral hemodynamics (van Everdingen et al., 1998; Kluytmans et al., 1999; Fabbri et al., 2014; Lin et al., 2015; Ren et al., 2015). For example, Kluytmans et al. (1999) found that in patients with a unilateral internal carotid artery occlusion, collateral flow via the ACoA is a sign of well-preserved cerebral hemodynamic status, whereas no collateral flow via the circle of Willis or flow via only the PCoA is a sign of deteriorated cerebral perfusion. In addition to directly modulating cerebral hemodynamics, the influence of ACoA and PCoA on the intrinsic ability of the cerebral vasculature to maintain cerebral hemodynamics also needs further study.

Cerebral autoregulation not only regulates cerebral hemodynamics, but also acts as a predictor for clinical occurrence and prognosis of several neurological disorders, such as cerebrovascular diseases (Guo et al., 2014a; Ma et al., 2016), Alzheimer’s disease (Claassen and Zhang, 2011), and patent foramen ovale (Guo et al., 2014b). Previous studies reported that collaterals may influence cerebral autoregulation (Reinhard et al., 2003a,c; Long et al., 2008). However, the extent of the influence of the ACoA and PCoA on cerebral autoregulation is not clear. Identifying the influence of collaterals on cerebral autoregulation both qualitatively and quantitatively may help us further understand the characteristics of cerebral vessels and help us select treatment strategies to improve cerebral hemodynamics. This is especially important in the management and secondary prevention of stroke in patients with stenosis in key cerebral arteries.

Accordingly, we determined the impact of the ACoAs and PCoAs on dynamic cerebral autoregulation (dCA) in a series of stroke patients with cerebral arterial stenosis/occlusion in our hospital. We found that the presence of ACoA/PCoA was associated with better dCA in the affected cerebral arteries and thus provided direct evidence for the important role of ACoA/PCoA during cerebral hemodynamics.

## Materials and Methods

The study design was approved by the ethics committee of the First Hospital of Jilin University. The data come from the departmental dCA database in the department of Neurology. All participants gave written informed consent.

### Participants

Non-acute ischemic stroke patients who underwent both dCA and digital subtraction angiography (DSA) examinations from September 2014 to July 2017 were searched in our dCA database. Inclusion criteria included: (1) either isolated basal artery and/or bilateral vertebral arteries severe stenosis/occlusion (group 1), or isolated unilateral internal carotid artery severe stenosis/occlusion (group 2) according to DSA by two neurologists. Exclusion criteria included: (1) acute phase of cerebral infarction (<15 days from onset); (2) received thrombolytic, interventional, or surgical treatment; and (3) other cerebral diseases (arterial aneurysm, intracranial tumors, and brain trauma). Patients in each group were further divided into subgroups according to presence or absence of ACoA or PCoA. The details of subgroups were as follows (**Table 1**):

**Table 1:** Baseline characteristics, phase difference, and gain values in each group.

	Male	Age (years)	Mean blood pressure (mmHg)	Right (affected) phase difference (degree)	Left (unaffected) phase difference (degree)	Right (affected) gain (cm/s/mmHg)	Left (affected) gain (cm/s/mmHg)
Group 1A (*n* = 11)	9 (81.8%)	58.09 ± 7.38	95.36 ± 8.91				
MCA	/	/	/	17.95 ± 6.10	20.68 ± 12.08	0.88 ± 0.38	1.11 ± 0.49
PCA	/	/	/	32.92 ± 10.54	34.09 ± 11.50	0.72 ± 0.32	0.76 ± 0.36
Group 1B (*n* = 10)	9 (90.0%)	62.20 ± 7.89	96.80 ± 11.07				
MCA	/	/	/	34.66 ± 12.23	35.23 ± 12.63	1.01 ± 0.28	1.20 ± 0.55
PCA	/	/	/	23.19 ± 10.15	24.85 ± 12.76	0.65 ± 0.34	0.71 ± 0.40
Group 2A (*n* = 9)	7 (77.8%)	55.89 ± 11.06	98.78 ± 8.93	20.95 ± 17.90	33.70 ± 17.67	0.66 ± 0.30	0.90 ± 0.44
Group 2B (*n* = 10)	10 (100%)	55.60 ± 13.66	93.10 ± 13.58	28.21 ± 6.13	35.07 ± 5.76	0.88 ± 0.33	0.76 ± 0.29
Group 2C (*n* = 11)	11 (100%)	53.18 ± 7.70	95.55 ± 10.42	14.94 ± 13.52	32.07 ± 7.73	0.79 ± 0.39	0.77 ± 0.48


**FIGURE 1:**
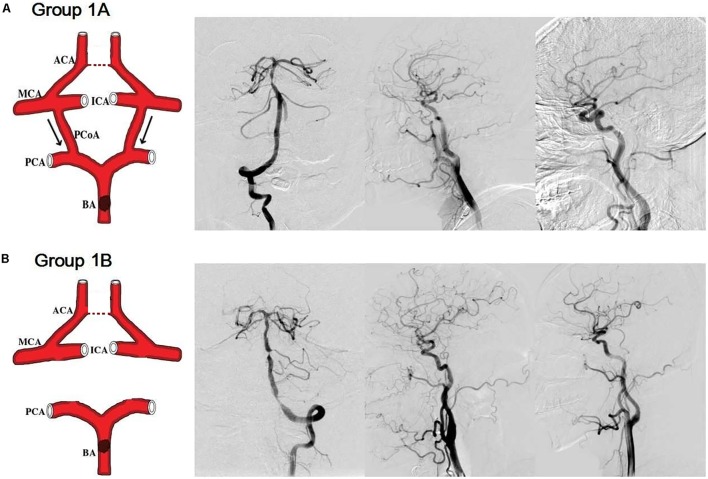
Simulation anatomic drawing, and representative digital subtraction angiography (DSA) manifestation of group 1A and group 1B. Severe stenosis of the basal artery, with/without anterior communicating artery, with cross-flow from anterior circulation to posterior circulation via the bilateral posterior communicating arteries (group 1A, **A**); without bilateral posterior communicating arteries (group 1B, **B**).

Groups 1A and 1B: (1) severe stenosis or occlusion of the basal artery and/or bilateral vertebral arteries; (2) without stenosis/occlusion of the internal carotid artery, the middle cerebral artery (MCA), and the anterior cerebral artery; (3) with/without ACoA; (4) with cross-flow from anterior circulation to posterior circulation via the bilateral PCoAs (group 1A, **Figure 1A**); without bilateral PCoAs (group 1B, **Figure 1B**).

**FIGURE 2:**
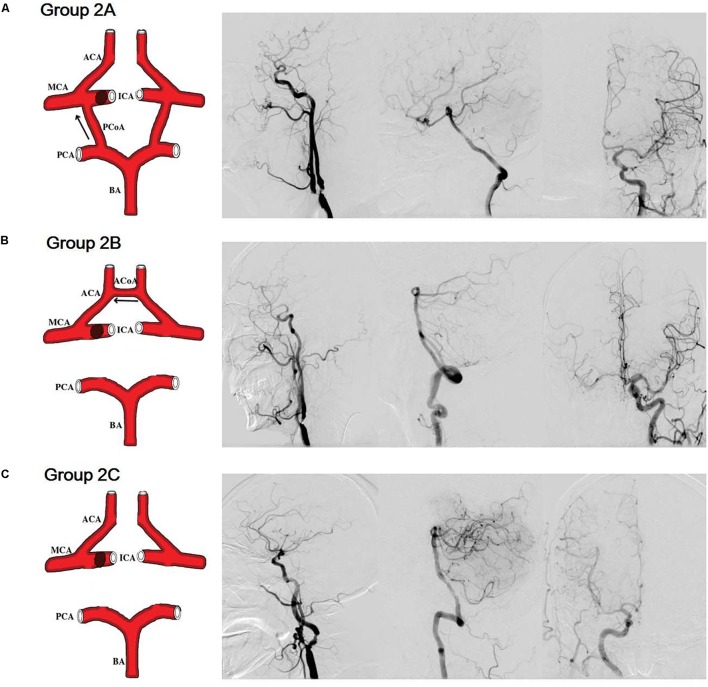
Simulation anatomic drawing, and representative digital subtraction angiography manifestation of group 2A, group 2B, and group 2C. **(A)** Severe stenosis of a unilateral internal carotid artery, without anterior communicating artery, with cross-flow from posterior circulation to anterior circulation via the posterior communicating artery of the affected side. **(B)** Severe stenosis of a unilateral internal carotid artery, without posterior communicating arteries, with cross-flow from the contralateral side to the affected side via the anterior communicating artery. **(C)** Severe stenosis of a unilateral internal carotid artery, without both anterior communicating artery and posterior communicating artery.

Group 2A: (1) severe stenosis or occlusion of a unilateral internal carotid artery; (2) without ACoA; (3) without stenosis/occlusion of the basal artery and/or vertebral artery; (4) with cross-flow from posterior circulation to anterior circulation via the PCoA of the affected side (**Figure 2A**).

Group 2B: (1) severe stenosis or occlusion of a unilateral internal carotid artery; (2) without PCoAs; (3) without stenosis/occlusion of the basal artery and/or vertebral artery; (4) with cross-flow from the contralateral side to the affected side via the ACoA (**Figure 2B**).

Group 2C: (1) severe stenosis or occlusion of a unilateral internal carotid artery; (2) without stenosis/occlusion of the basal artery and/or vertebral artery; (3) without both ACoA and PCoA (**Figure 2C**).

### DCA Examination

For stenosis/occlusion of the basal artery and/or bilateral vertebral arteries, we conducted the dCA examination from both the bilateral middle cerebral arteries and bilateral posterior cerebral arteries. For stenosis/occlusion of the internal carotid artery, we conducted the dCA examination only from the bilateral middle cerebral arteries.

This method was reported previously (Guo et al., 2015; Ma et al., 2016). Briefly, continuous cerebral blood flow velocity was recorded non-invasively using transcranial Doppler (MultiDop X2, DWL, Sipplingen, Germany) in the bilateral middle cerebral artery at a depth of 45 to 60 mm and the bilateral posterior cerebral artery at a depth of 60 to 70 mm. Spontaneous arterial blood pressure was recorded using a servo-controlled plethysmograph (Finometer PRO, Netherlands) on one middle finger. Continuous data were recorded for 5–10 min and then were stored for further dCA analysis.

### DCA Analysis

The analog output of the continuous arterial blood pressure measurement device was plugged into the A/D convertor of the transcranial Doppler device for recording the digitized arterial blood pressure and cerebral blood flow velocity simultaneously. Two digital signals were then aligned to remove the time lags calculated by a cross-correlation function. In this study, with all recordings less than 10 min, we did not find any evident data drifting after the alignment. The dCA analysis was performed as previously reported using MATLAB (MathWorks, Natick, MA, United States) (Guo et al., 2015; Ma et al., 2016). Briefly, beat-to-beat alignment of the data was achieved with a cross-correlation function to eliminate possible time lags. The relationship between dynamic changes in spontaneous arterial blood pressure and bilateral middle cerebral artery blood flow/bilateral posterior cerebral artery blood flow was assessed with a transfer function analysis (Claassen et al., 2016). For each recording, arterial blood pressure and bilateral cerebral artery blood flow velocity were divided into a number of data segments by a 60-s window with 30-s overlap. For one segment of arterial blood pressure and bilateral cerebral artery blood flow velocity, the transfer function analysis was implemented as,

$$H(f)=\frac{S_{pv}\left(f\right)}{S_{pp}\left(f\right)}$$

where *H*(*f*) denotes the frequency response. *S_pp_*(*f*) is the auto-spectrum of arterial blood pressure and *S_pv_*(*f*) is the cross-spectrum between arterial blood pressure and cerebral artery blood flow velocity. For each subject, *S_pp_*(*f*) and *S_pv_*(*f*) were averaged over the segments to improve statistical reliability. The phase difference *ϕ*(*f*) can then be computed as,

$$\phi (f)=\tan^{-1}\left[\frac{H_{I}\left(f\right)}{H_{R}\left(f\right)}\right]$$

where *H_R_*(*f*) and *H_I_*(*f*) are the real and imaginary parts of *H*(*f*), respectively. In the frequency domain, because previous studies of dCA suggested that autoregulatory parameters in a low frequency band (0.06–0.12 Hz) is more meaningful than in the other frequency bands (Reinhard et al., 2003b, 2004), thus the phase difference and coherence function within 0.06–0.12 Hz were estimated to evaluate dCA. A coherence threshold of >0.4 was chosen to define a lower limit of the linearity between arterial blood pressure and cerebral blood flow velocity so as to apply the transfer function analysis (Zhang et al., 1998).

### Statistical Analysis

The Statistical Package for the Social Sciences Version 17.0 (SPSS, IBM, West Grove, PA, United States) was used to analyze the data. Continuous data are expressed as mean ± standard deviation and were analyzed using Student’s *t*-tests. The discrete variables are expressed as the rate (percentage) and were analyzed using chi-squared and Fisher’s exact tests. *P*-values < 0.05 were considered statistically significant.

## Results

### Participant Characteristics

A total of 231 patients who underwent both dCA and DSA examinations were identified in the departmental database. Most of these patients had a combination of multiple cerebral vascular stenosis and thus were excluded. A subtotal of 51 patients were selected, including 21 patients in the group 1, and 30 patients in the group 2. The group 1A included 11 patients; group 1B included 10 patients; group 2A included 9 patients; group 2B included 10 patients; group 2C included 11 patients.

There were no significant differences in gender, age, and mean blood pressure between group 1A and group 1B (**Table 1**, *P* > 0.055; **Supplementary Data Sheet S1**), and among group 2A, group 2B, and group 2C (**Table 1**, all *P* > 0.05; **Supplementary Data Sheet S1**).

### Protective Effect of PCoAs on dCA in Patients With Isolated Basal Artery and/or Bilateral Vertebral Artery Severe Stenosis/Occlusion

**FIGURE 3:**
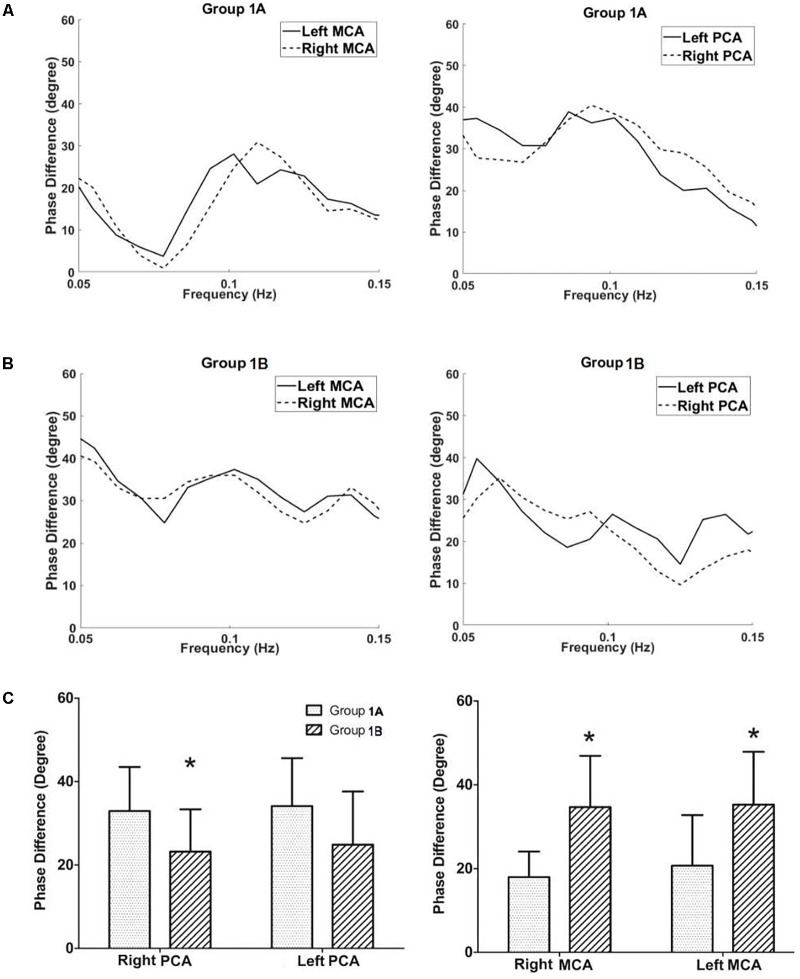
Dynamic cerebral autoregulation analysis in subgroups of groups 1. The autoregulatory parameter (phase difference) of the bilateral middle cerebral arteries and bilateral posterior communicating arteries derived from the transfer function in group 1A **(A)**, and group 1B **(B)**. **(C)** Statistical analysis of groups 1A and 1B. Left half of **(C)**: the phase difference values of the right posterior cerebral arteries in group 1A were significantly higher than those at the same side of group 1B. The phase difference values of the left posterior cerebral arteries in group 1A tended to be higher compared with group 1B, although the value was not statistically significant. Right half of **(C)**: the phase difference values of the bilateral middle cerebral arteries in the group 1A were significantly lower than those at the corresponding side of the group 1B. ^∗^*P* < 0.05 for comparison with group 1A.

In Group 1, patients with severe stenosis/occlusion of the basal artery and/or bilateral vertebral arteries, the phase difference values (autoregulatory parameter) of the right posterior cerebral artery in group 1A (with PCoAs) were significantly higher than those of right posterior cerebral artery in group 1B (without PCoAs, *P* = 0.045, **Figure 3**). The phase difference values of the left posterior cerebral arteries in group 1A tended to be higher compared with group 1B, although the *P*-value was not statistically significant (*P* = 0.097, **Figure 3**).

On the contrast, the phase difference values of the bilateral middle cerebral arteries in the group 1A were significantly lower than those at the corresponding side of the group 1B (right: *P* = 0.001; left: *P* = 0.014; **Figure 3** and **Table 1**). There was no difference in gain values (another autoregulatory parameter) between group 1A and group 1B in both posterior cerebral arteries and middle cerebral arteries.

### Protective Effect of ACoA or PCoA on dCA in Patients With Isolated Unilateral Internal Carotid Artery Severe Stenosis/Occlusion

In Group 2, patients with isolated unilateral internal carotid artery severe stenosis/occlusion, the phase difference values in the middle cerebral artery of the affected side in group 2A (with PCoA, but without ACoA) tended to be lower compared with the group 2B (with ACoA, but without PCoA, *P* = 0.275) whereas they tended to be higher compared with the group 2C (without both ACoA and PCoA, *P* = 0.403), though neither of these was statistically significant.

**FIGURE 4:**
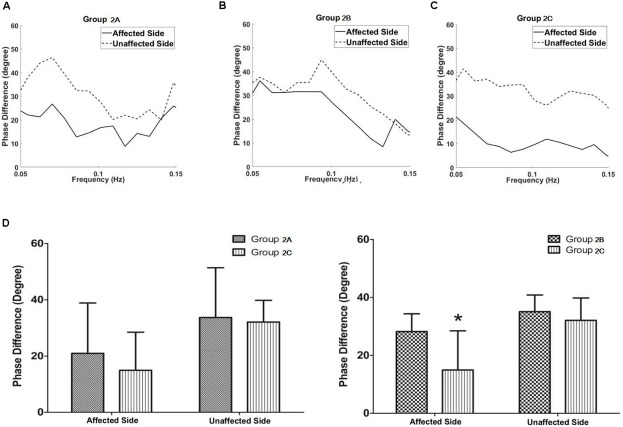
Dynamic cerebral autoregulation analysis in subgroups of groups 2. The autoregulatory parameter (phase difference) of the bilateral middle cerebral arteries derived from the transfer function in group 2A **(A)**, group 2B **(B)**, and group 2C **(C)**. **(D)** Statistical analysis of groups 2A and 2C; and groups 2B and 2C. Left half of **(D)**: the phase difference values in the middle cerebral artery at the affected side in group 2A tended to be higher compared with the group 2C, although the value was not statistically significant. Right half of **(D)**: the phase difference values in the middle cerebral artery at the affected side in the group 2B were significantly higher than those in the group 2C. ^∗^*P* < 0.05 for comparison with group 2C.

The phase difference values in the middle cerebral artery of the affected side in the group 2B were significantly higher than those in the group 2C (*P* = 0.011, **Figure 4** and **Table 1**). The phase difference values of the unaffected side were similar in each group. There was no difference of gain values in each group.

## Discussion

In this study, combining dCA and DSA in non-acute stroke patients with posterior circulation arteries/a unilateral internal carotid artery severe stenosis/occlusion, we found the cross-flow of the ACoA/PCoA to the affected area appears to compensate for potentially compromised dCA in the affected area via providing blood flow. These findings suggest an important role of the ACoA/PCoA in dCA regulation.

The dCA analysis reveals information about the intrinsic ability of the cerebral vasculature to maintain sufficient blood supply, which might be a more appropriate method for evaluating cerebral hemodynamic impairment (Reinhard et al., 2003a). Pathophysiologically, insufficient collateral blood supply leads to a reduced perfusion pressure in the area downstream of the stenosis/occlusion site, causing dilation of downstream cerebral arterioles, and resulting in the impairment of dCA (Reinhard et al., 2003a). In the present study, we found that in cases of severe stenosis/occlusion of the basal artery and/or bilateral vertebral arteries, the dCA of the posterior cerebral artery (PCA) was higher in patients with PCoA than those without PCoA. This may be because sufficient blood flow results from anterior to posterior circulation via the PCoA to compensate for impaired posterior circulation. However, this may cause relatively insufficient blood supply within the anterior circulation, as reflected by the lower dCA of the middle cerebral artery in the presence of PCoA. When the middle cerebral artery and its branches are dilated in response to dCA, the remaining dCA in the middle cerebral artery is lower.

In patients with severe stenosis/occlusion of a unilateral internal carotid artery, we found the dCA of the middle cerebral artery in the affected side was significantly better in patients with ACoA than those without ACoA. This is consistent with cross-flow compensation of anterior communicating artery from the unaffected to the affected side. Kluytmans et al. (1999) found that in patients with a unilateral internal carotid artery occlusion, collateral flow via the ACoA is a sign of well-preserved hemodynamic status (Kluytmans et al., 1999). Our findings support their results, as well as the results of Reinhard et al. (2003a,c). Their studies found that in patients with severe unilateral carotid stenosis/occlusion with ACoA, dCA at the affected side did not differ significantly from the dCA at the contralateral side. All these studies demonstrated an important role of the ACoA in regulating the dCA. In addition, in group 2 patients, we found that the dCA tended to be higher among patients with PCoA compared to those without PCoA, although the difference did not reach statistical significance probably due to low numbers of patients. We considered this phenomenon may be due to the compensatory cross-flow from posterior circulation to anterior circulation through PCoA. It’s worth mentioning that, following the development of 3D computational modeling, other groups also found the potential important role of circle of Wills on cerebral autoregulation (Moore et al., 2005; Long et al., 2008).

Though not every patient possesses integrated collateral circulation, it’s shown that the communicating arteries play important roles in maintaining adequate perfusion status during ischemic demand (van Seeters et al., 2015; Goksu et al., 2017).

Our study here suggested a regulatory role of communicating arteries on dCA, which subsequently may modulate the occurrence and outcomes of ischemic stroke. Furthermore, our findings increased understanding of the cerebral hemodynamic characteristics in stroke patients with and without communicating arteries.

The study has several limitations which may have compromised the internal and external validity. Firstly, this study was a retrospective not prospective study. While we sought to exclude stroke patients in acute phase, the post-stroke time range was relatively wide, from 15 days to 2 months. Thus, we cannot completely exclude the time-course effects of the disease. Secondly, the statistical power of our findings was limited by the relatively small sample size of our study, which also possibly caused that the difference of several subgroups did not reach statistical significance. Thirdly, our study is based on our experience within one large tertiary care center in one province of China. The extent to which our results could be generalized to other different or broader patient populations is therefore unknown.

## Conclusion

The present study suggests that cross-flow of the ACoA/PCoA to areas affected by severe stenosis and/or occlusion can compensate for compromised dCA in that area, consistent with an important protective function of the ACoA/PCoA in dCA compensation.

## Availability of Data and Materials

All datasets or this study are included in the manuscript and the supplementary files.

## Author Contributions

YY and HJ conceived and overseed study. HS, YZ, ZW, XY, XS, CL, and HJ performed data collection. JL and HM performed data analysis. Z-NG and PZ performed statistical analysis. BX and HZ read the DSA. Z-NG and XS wrote manuscript.

## Conflict of Interest Statement

The authors declare that the research was conducted in the absence of any commercial or financial relationships that could be construed as a potential conflict of interest.

## Supplementary Material

DATA SHEET S1 Gender, age, mean blood pressure and phase difference in each group.
